# Priapism Presentations in a Saudi Arabian Emergency Department: A Retrospective Study at a Tertiary Care Hospital

**DOI:** 10.3390/healthcare12171716

**Published:** 2024-08-28

**Authors:** Baraa Alghalyini, Abdul Rehman Zia Zaidi, Kanza Atif, Noorah Mosharraf, Hala Tamim, Muhammad Nauman Qureshi

**Affiliations:** 1 Department of Family and Community Medicine, College of Medicine, Alfaisal University, Riyadh 11533, Saudi Arabia; htamim@yorku.ca; 2 Department of Emergency Medicine, King Faisal Specialist Hospital and Research Center, Riyadh 11564, Saudi Arabia; qmuhammad@kfshrc.edu.sa; 3 College of Medicine, Alfaisal University, Riyadh 11533, Saudi Arabia; katif@alfaisal.edu (K.A.); nouram2010@hotmail.com (N.M.); 4 School of Kinesiology and Health Science, York University, Toronto, ON M3J 1P3, Canada

**Keywords:** priapism, proportion, Saudi Arabia, retrospective study, emergency department

## Abstract

Objectives: To examine the distribution, clinical characteristics, and management of priapism in a Saudi Arabian tertiary care setting to provide a regional perspective. Subjects and Methods: This retrospective chart review included 29 male patients presenting with priapism at a tertiary care hospital in Riyadh, Saudi Arabia, from January 2011 to June 2023. Data were collected on patient demographics, clinical presentation, treatment modalities, and outcomes. Results: The study found recurrent episodes of priapism in many patients, with a significant number associated with hematological diseases, notably sickle cell disease. Most treatments involved non-surgical methods. A notable finding was the correlation between the duration of priapism episodes and the likelihood of hospital admissions, suggesting that prolonged episodes often required more extensive medical attention. Conclusions: Priapism often presents as a chronic and recurrent condition requiring personalized management strategies. This study emphasizes the importance of recognizing regional occurrence patterns to enhance the management of priapism and suggests a need for further research in regions where this condition is less common.

## 1. Introduction

Priapism is defined as a “persistent, often painful penile erection and an emergency unrelated to sexual stimulation” [1,2,3]. This condition represents a urological emergency that requires prompt diagnosis and management to prevent long-term complications, including erectile dysfunction [4]. Its complex pathophysiology is due to the disrupted regulation of blood perfusion within the penis. Priapism can be divided into three types:

- Ischemic (low-flow) priapism: This is the most commonly occurring and time-sensitive condition that involves blood vessels becoming obstructed within the penis. It is crucial that this issue is treated promptly as failure to do so may result in the individual experiencing permanent erectile dysfunction (ED) [5].
- Non-ischemic (high-flow) priapism: This form is caused by unregulated arterial blood flow into the corpora cavernosa without associated venous trapping, often resulting from trauma. Unlike ischemic priapism, patients usually do not develop tissue ischemia, and this condition is typically painless [5,6,7].
- Recurrent (stuttering) priapism: This is characterized by periodic episodes of prolonged erections that may lead to recurrent ischemic priapism and is usually associated with sickle cell disease [8,9].

The origin of priapism has been documented in the medical literature for a considerable amount of time. However, determining its prevalence is challenging, with an estimated incidence of 1.1–1.5 cases per 100,000 person-years [4]. Common causes of priapism can range from blood abnormalities to structural deformities [8,9]. Sickle cell disease, treatment of erectile dysfunction, perineal trauma, diabetes, tumors of urological organs, and medications such as antidepressants, antihypertensives, and anticoagulants are well-known risk factors [5,10]. The development of fibrous tissue in the penile tissue, characterized by the significant obstruction of the vascular system, is the root cause of this issue. This leads to erectile dysfunction, making prompt treatment a crucial necessity.

Priapism is identified through the amalgamation of three elements: the patient’s medical history, physical examination, and analysis of blood gas levels extracted from the corpora cavernosa. Acidosis and altered oxygen/carbon dioxide levels signal the ischemic type, while arterial-like gas readings suggest the non-ischemic form. To determine the cause and assess the viability of tissues, various technologies, including blood tests, coagulation studies, Doppler ultrasound, and MRI, are used to pinpoint the cause and assess tissue viability [6,8].

The treatment objectives are to reverse engorgement (detumescence) and relieve pain, while simultaneously preserving erectile function. Ischemic priapism constitutes a medical emergency. Penile blood aspiration and intracavernous injections (usually phenylephrine) are the first line of defense and are labelled as non-invasive treatments. Invasive treatment is surgical shunting which is used when the first two are unsuccessful in achieving detumescence. The non-ischemic type may initially warrant observation for spontaneous resolution [8]. Selective arterial embolization is preferred for persistent cases, and surgery is a last resort. Recurring priapism typically employs similar acute treatment approaches, with an emphasis on prevention through medications, such as gabapentin, digoxin, GnRH agonists, estrogens, and antiandrogens. However, the potential side effects of these medications must be carefully considered and managed [8,9,10,11].

When managing priapism, both invasive and non-invasive approaches play crucial roles. The chosen method hinges on the type of priapism, its duration, and the patient’s response to initial care. Non-invasive approaches include the following:

- Penile blood aspiration: Often the first step for ischemic priapism, this involves draining stagnant blood from the penis to reduce internal pressure and restore normal blood flow [3].
- Intracavernous injections: These typically use drugs like phenylephrine to constrict blood vessels and alleviate swelling [3,6,8].
- Conservative management: For non-ischemic priapism, which often resolves on its own, ice packs and observation are common practices [3,6].

Invasive approaches include the following:

- Surgical drainage: Considered when non-invasive methods fail; procedures like Winter, Al Ghorab, or Grayhack shunts create a pathway for blood to exit the penis, reducing pressure.
- Penile implant: For persistent or recurring ischemic priapism, a penile implant might be used to maintain function and length.

The decision between invasive and non-invasive care is critical and time sensitive. Recent advancements in imaging, like the color Doppler ultrasound, have improved our ability to distinguish between ischemic and non-ischemic priapism, guiding treatment choices [9,10]. Furthermore, using point-of-care ultrasound in emergencies has been proven effective in identifying non-ischemic priapism, potentially avoiding unnecessary invasive procedures [6,9,11].

In children, especially those with blood disorders, there is an ongoing debate about the need for immediate surgery. Some research suggests that a conservative approach with blood transfusions and proper hydration might be successful, potentially reducing the risks of invasive treatments in this vulnerable group [4,10,12].

In emergency settings, diagnosing priapism can be challenging due to various factors. These include patient embarrassment leading to delayed presentation, the need for rapid differentiation between ischemic and non-ischemic types, and the potential for misdiagnosis in cases of recurrent or stuttering priapism. Timely and accurate diagnosis is crucial, as prolonged ischemic priapism can lead to irreversible tissue damage and erectile dysfunction [10,11].

The current understanding, relying primarily on case reports and the findings of small studies, lacks comprehensive insights into large-scale investigations. Moreover, data from diverse clinical settings are limited but are crucial for developing the most effective, context-specific treatment practices [10,11].

The diverse manifestations and multifarious risk factors associated with priapism necessitate an individualized approach in certain populations. In Saudi Arabia, the distinctive characteristics of the population and the healthcare system present a unique opportunity to investigate patterns of priapism, potentially uncovering regional differences in prevalence, comorbidities, and treatment efficacy [12].

### 1.1. Problem Statement

A significant gap exists in our understanding of how best to manage priapism. This is because many studies have failed to capture a comprehensive understanding of priapism, owing to their limited scope. Additionally, the scarcity of statistically robust data on individuals from diverse communities hampers researcher ability to develop evidence-based practices that consider geographic and cultural differences.

### 1.2. Significance of the Study

By mapping cases of priapism in the Saudi tertiary care setting, our research seeks to fill the gaps in the current literature. Our objective is to describe demographic parameters, disease presentation, treatment modalities, and results in our setting. This information may be utilized to devise tailored approaches for addressing priapism in emergency situations and guide future investigative initiatives.

### 1.3. Aims and Objectives

This retrospective study examined priapism cases at the King Faisal Specialist Hospital & Research Centre in Riyadh over a 12-year period to perform the following:

1. Determine the proportion of priapism cases across different age groups and regions within Saudi Arabia;
2. Investigate the correlation between priapism and underlying medical conditions, particularly blood disorders, including sickle cell disease;
3. Evaluate the role of non-invasive and invasive treatment of priapism in achieving detumescence;
4. Explore potential connections of priapism with factors like prior history, triage assessment, pain scores, detumescence outcomes, and the length of hospital stays.

## 2. Methods

### 2.1. Study Design and Setting

This retrospective chart review was conducted at the King Faisal Specialist Hospital & Research Centre (KFSH), a research and referral center in Riyadh, Saudi Arabia. KFSH is a renowned tertiary care hospital that has gained recognition for its medical services and research accomplishments in the region. The study specifically focused on priapism cases presented to the Emergency Medicine (EM) Department, a primary contact point for patients with acute medical conditions. This setting provided access to diverse patient populations and comprehensive medical records through the electronic medical record (EMR) system.

### 2.2. Study Population

The target population included male patients who were diagnosed with priapism. Past records from 1 January 2011 to 1 June 2023 were examined. Initially, 35 patients were identified with a diagnosis of priapism. However, 6 patients were excluded due to incomplete medical records, potentially compromising data integrity. The final study population consisted of 29 patients to strengthen the reliability of the study findings.

The inclusion criteria were the following:

- Male patients of any age presented to the ER department;
- Diagnosis of priapism confirmed by a urologist;
- Treatment received at the KFSH, Riyadh.

The exclusion criteria were the following:

- Incomplete medical records lacking essential management details;
- Patients who left against medical advice before the completion of initial treatment;
- Cases where the final diagnosis was changed to a condition other than priapism.

### 2.3. Quality Assurance

The relatively low incidence of priapism in the institution’s EMR necessitated data collection by a single abstractor. To ensure the consistency and accuracy of the review process and its results, the abstractor was trained in using a standardized chart review procedure, variables of interest, and methods for identifying and classifying priapism.

### 2.4. Data Collection

Meticulous protocols and guidelines were followed by the abstractor to electronically extract data from medical records for the study with maximum accuracy and dependability. Data from the KFSH Emergency Medicine Department were systematically and uniformly extracted in accordance with standardized procedures, with the objective of identifying the most crucial aspects, such as demographics (e.g., age, regional distribution), medical history, predisposing conditions (e.g., sickle cell disease, urological diseases), medication history, laboratory test results (e.g., hemoglobin, reticulocyte count), imaging findings (e.g., ultrasound, MRI), priapism clinical characteristics (e.g., duration, severity, and recurrence), treatment (pharmacological and surgical interventions), and treatment outcomes (e.g., detumescence/resolution, hospital admission).

The independent variables of this study were priapism duration, patient age, regional distribution, hematological causes, medications, types of priapism, and initial treatments. The dependent variables were hospital admission, invasive and non-invasive procedures, erectile dysfunction, penile fibrosis, and disease recurrence. Penile fibrosis was assessed through a combination of physical examination and penile ultrasound, which were used to detect fibrotic changes in the corpora cavernosa. Priapism was classified based on the duration of erection, with ischemic priapism typically defined as an erection lasting more than 4–6 h, beyond which the risk of erectile dysfunction increases significantly. The classification criteria were as follows:

- Ischemic priapism: Painful, rigid erection lasting >4 h with little or no cavernous blood flow.
- Non-ischemic priapism: Painless, partially rigid erection with normal to high cavernous blood flow.
- Recurrent (stuttering) ischemic priapism: Intermittent episodes of ischemic priapism, often associated with sickle cell disease [2,3,5,6,7].
- Stringently adhering to these protocols enabled extensive data collection, essential for thorough analysis and gaining valuable insights into the management of priapism in emergency settings.

### 2.5. Statistical Analysis

Data were analyzed using SPSS version 26.0 (IBM Corp., Armonk, NY, USA). Continuous variables were presented as mean ± standard deviation or median (interquartile range IQR) based on the distribution of data. Categorical variables were presented as frequencies and percentages. Descriptive statistics were used to summarize patient characteristics, priapism types, and treatment outcomes. Advanced statistical techniques, including logistic regression analysis, were used to explore the associations between variables, identify significant risk factors, and examine correlations between clinical parameters and treatment outcomes. Chi-square was used for categorical variables, and t-tests were used for continuous variables. A *p*-value < 0.05 was considered statistically significant. This approach facilitated pattern identification and revealed predictors relevant to the management of priapism for evidence-based clinical decision making.

### 2.6. Ethical Considerations

This study adhered to the highest ethical standards and human research guidelines. Ethics approval was received from the Office of Research Affairs, Research Advisory Council, and the Institutional Review Board of the King Faisal Specialist Hospital & Research Centre (approval # 2231192). Strict measures were implemented to de-identify data during the collection phase so that patient privacy was preserved, and the ethical principles of beneficence, non-maleficence, autonomy, and justice were respected. The need for informed consent was waived due to the retrospective nature of the study and the use of de-identified data.

## 3. Results

### 3.1. Participant Demographics

The study included 29 Saudi male patients diagnosed with priapism. The mean age was 36.59 years (SD = 15.67), with a range from 13 to 79 years and a median age of 31 years (IQR: 27–43 years). The age distribution analysis is detailed in Table 1.

The mean age of participants was 36.59 years (95% CI: 30.63–42.55 years). A one-sample Kolmogorov–Smirnov test was conducted to assess the normality of age distribution (D = 0.145, *p* = 0.121), indicating that the age distribution did not significantly deviate from normality.

The regional distribution of patients generally aligned with the population density across Saudi Arabia, as seen in Table 2. This distribution reflects the population density trends in Saudi Arabia, showcasing the spread of patients across different regions. However, it is important to note that this study’s retrospective nature and single-center design limit the ability to make assumptions about the overall incidence of priapism in the Saudi population. The distribution reflects population density trends in Saudi Arabia, showing the spread of patients in different regions of Saudi Arabia.

### 3.2. Comorbidities and Medical History

In our study population, we identified several comorbidities and risk factors associated with priapism as shown in Table 3. The average number of recurrent episodes per patient was 3.5 (range: 2–6). All recurrent episodes occurred in different patients, and in each case, the type of priapism remained consistent across episodes.

The analysis of patients with single versus multiple risk factors is shown in Table 4.

Among patients with multiple risk factors, the most common combinations are shown in Table 5.

These findings highlight the complex interplay of various risk factors in the development of priapism. Hematological disorders, particularly sickle cell disease, emerged as the predominant risk factor. The presence of multiple risk factors in 31% of patients highlights the multifactorial nature of priapism etiology in many cases.

### 3.3. Types of Priapism

**Figure 1:**
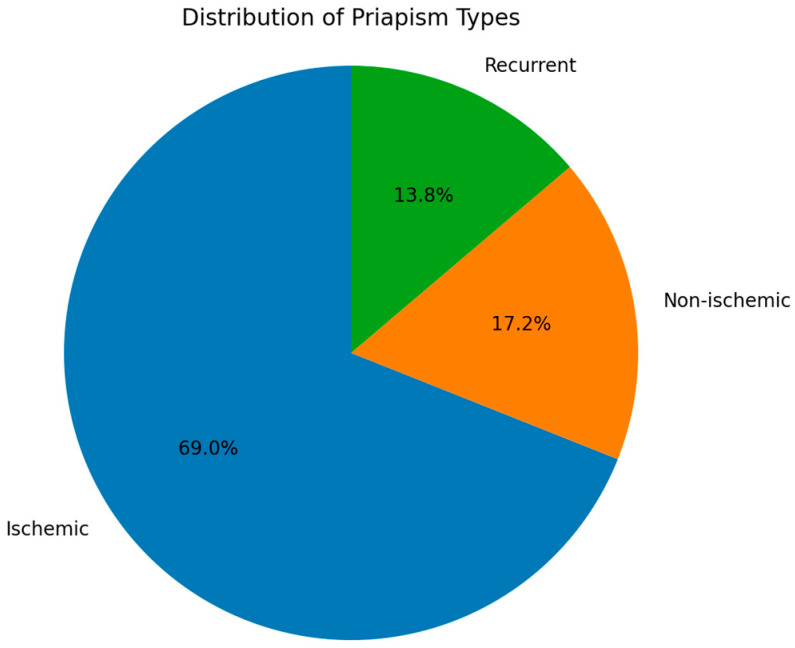
Distribution of priapism types.

Our analysis of priapism cases revealed distinct patterns in the distribution of priapism types. Ischemic priapism was the most prevalent form, accounting for 20 patients or 69.0% of the cases. Non-ischemic priapism was observed in five patients, representing 17.2% of the cases. The least common type was recurrent priapism, seen in four patients, which constituted 13.8% of the cases. These findings highlight the predominance of ischemic priapism in our study population, while also indicating the presence of less common forms of the condition. The types of priapism observed in our study population are seen in Table 6 and Figure 1.

### 3.4. Relationship between Risk Factors and Priapism Types

1. Ischemic priapism: Among the 20 patients with ischemic priapism, 11 (55%) had sickle cell disease as a primary risk factor. Cardiovascular risk factors were present in six (30%) of the ischemic priapism cases.
2. Non-ischemic priapism: Of the five patients with non-ischemic priapism, two (40%) had a history of pelvic trauma, while one (20%) had Peyronie’s disease. The remaining two cases had no identifiable risk factors.
3. Recurrent priapism: All four patients with recurrent priapism had underlying hematological disorders: three (75%) with sickle cell disease and one (25%) with thalassemia.

**Figure 2:**
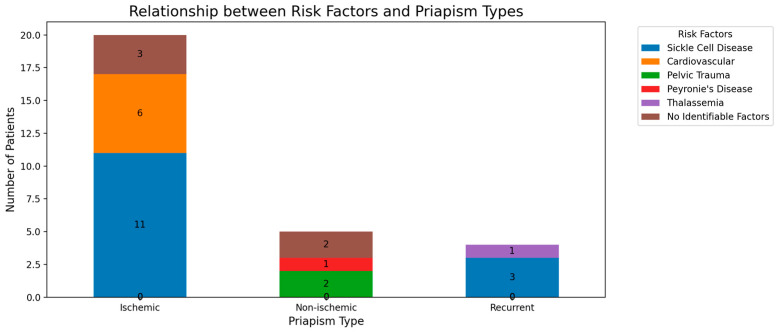
Relationship between risk factors associated with priapism types.

These findings suggest a strong association between hematological disorders, particularly sickle cell disease, and both ischemic and recurrent priapism. Non-ischemic priapism appears to have a more diverse etiology in our study population. The relationship can be seen in Figure 2.

A chi-square test of independence was performed to examine the relationship between priapism types and risk factors. The relation between these variables was significant, χ^2^(8, N = 29) = 18.73, *p* = 0.016. Post-hoc analysis using standardized residuals revealed that sickle cell disease was significantly associated with ischemic priapism (z = 2.41, *p* < 0.05). The percentage of patients with each comorbidity by priapism type is shown in the stacked bar chart (Figure 2).

### 3.5. Clinical Presentation and Management

Our study of 29 patients with priapism revealed significant insights into treatment efficacy and patient outcomes (Table 7). We observed three types of priapism: ischemic, non-ischemic, and recurrent. We prioritized non-surgical interventions initially, aligning with established guidelines. Non-invasive procedures were attempted in all 29 patients, with varying degrees of success as shown in Table 1. Out of the 29 patients, 22 had ischemic priapism, 3 had non-ischemic priapism, and 4 had recurrent priapism.

Penile aspiration proved effective in 10.3% of the cases, while intracavernous injections successfully resolved priapism in 44.8% of the patients. Conservative management was successful in 3.4% of the cases. The overall success rate for non-invasive procedures was 58.6% (17/29 patients).

**Figure 3:**
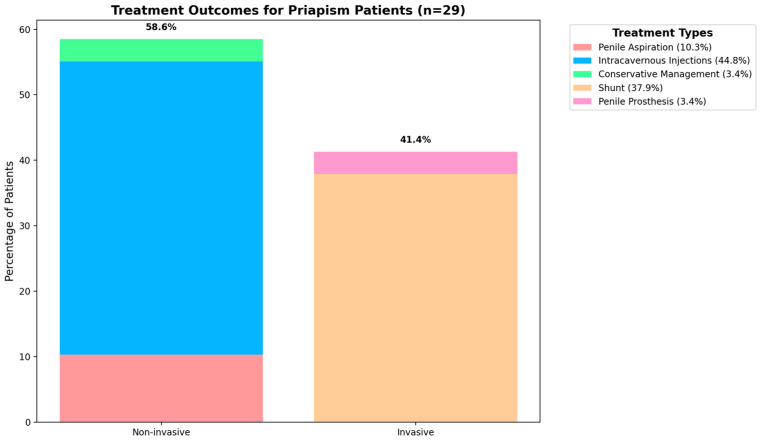
Treatment outcomes for priapism patients.

For the remaining 41.4% of patients who did not respond to non-invasive treatments, invasive procedures were necessary. As detailed in Table 1, shunt procedures were performed on 37.9% (11/29) of the patients, while penile prosthesis implantation was required for 3.4% (1/29). Notably, the success rate for invasive procedures was 100%, effectively resolving priapism in all cases where non-invasive methods had failed. This can be seen in Figure 3.

Patient presentation timing played a crucial role in treatment outcomes. As shown in Table 2, the mean symptom duration varied among ischemic, non-ischemic, and recurrent priapism cases. Our study reported varying rates of complete resolution for each type, underscoring the importance of tailored interventions (Table 8).

**Figure 4:**
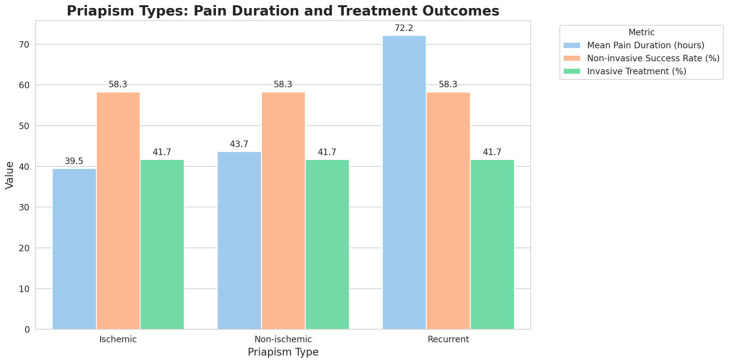
Priapism types: Pain duration and treatment outcomes. Grouped bar chart showing the Mean Pain Duration, Non-invasive Success Rate, and Invasive Treatment percentage for each Priapism Type (Ischemic, Non-ischemic, and Recurrent).

Statistical analyses provided further insights into the relationship between priapism type and pain duration. A one-way ANOVA analysis, summarized in Table 2, showed a significant effect of priapism type on pain duration. Post-hoc comparisons indicated significant differences in mean pain duration among the three types of priapism. See Figure 4, which shows the mean pain duration, non-invasive success rate, and invasive treatment percentage for each priapism type (ischemic, non-ischemic, and recurrent).

Logistic regression analysis, presented in Table 3, revealed that pain duration and priapism type were significant predictors of the likelihood of requiring invasive treatment. The model explained 52.1% (Nagelkerke R^2^) of the variance in treatment type and correctly classified 79.3% of the cases (Table 9).

For patients with recurrent priapism (n = 4), the average number of episodes was 3.5 (range: 2–6) over a 12-month period. All recurrent cases were of the ischemic type in our study sample. The occurrence of complications, particularly erectile dysfunction, emphasizes the need for long-term follow-up and management strategies for these patients.

### 3.6. Outcomes for Sickle Cell Patients

Table 10 shows the treatment and outcomes in patients with sickle cell disease.

### 3.7. Triage Levels

**Figure 5:**
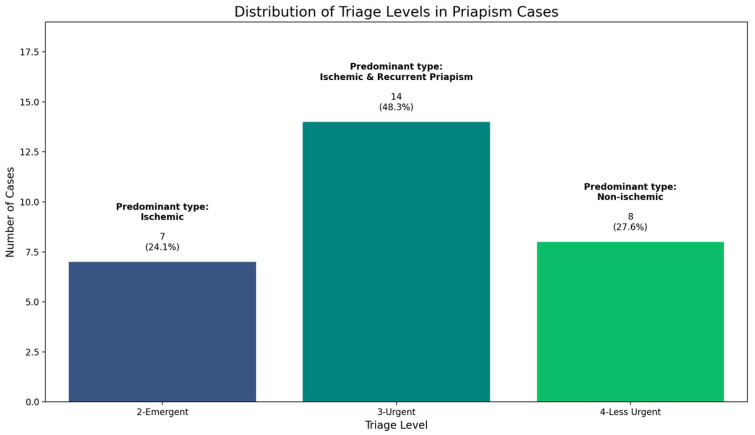
Distribution of triage levels in priapism cases (n = 29).

An analysis of 29 priapism cases revealed a distinct distribution of triage levels, providing insight into the urgency of care required. The majority of cases (48.3%, n = 14) were classified as 3-Urgent, followed by 4-Less Urgent (27.6%, n = 8) and 2-Emergent (24.1%, n = 7). This distribution correlated with priapism types, with 2-Emergent cases predominantly associated with ischemic priapism, reflecting the time-sensitive nature of this condition. The 3-Urgent category included a mix of ischemic and stuttering priapism, highlighting the variability in presentation. Most 4-Less Urgent cases were associated with non-ischemic priapism, consistent with its generally less severe nature as seen in Figure 5. In this study, ‘2-Emergent’ refers to cases requiring immediate medical intervention within minutes, typically due to the risk of irreversible damage, while ‘Less Urgent’ cases were those where immediate intervention was not critical, though treatment was still needed within hours.

The observed success rates provide valuable information for clinical decision-making, suggesting that a significant proportion of priapism cases can be effectively managed with non-invasive techniques. However, the substantial percentage requiring invasive treatment highlights the need for a comprehensive approach to priapism management, with the capability to escalate to more aggressive interventions when necessary.

### 3.8. Acute Management and Long-Term Follow-Up Interventions

#### 3.8.1. Acute Management: Blood Transfusions

In the acute management phase, four patients (15.4% of the total cohort) required blood transfusions.

#### 3.8.2. Long-Term Follow-Up Management

The long-term follow-up treatment consisted of two main interventions:

(a) Bone marrow transplantation: Performed in four patients (15.4% of the total cohort).
(b) Penile prosthesis insertion/removal: Conducted in seven patients (26.9% of the total cohort).

### 3.9. Prior Priapism History and Shunt Surgery

This analysis revealed an unusual trend: 8.7% of the patients without previous priapism underwent emergency shunt surgery, but none of the patients with previous history did. This indicates that priapism events may not necessarily be directly linked to the requirement for emergency shunt surgery. This result might also suggest the success of continued management approaches in preventing severe recurrences which would require surgical intervention.

### 3.10. Correlation between Priapism Duration, Hospital Admission, and Treatment Outcomes

**Figure 6:**
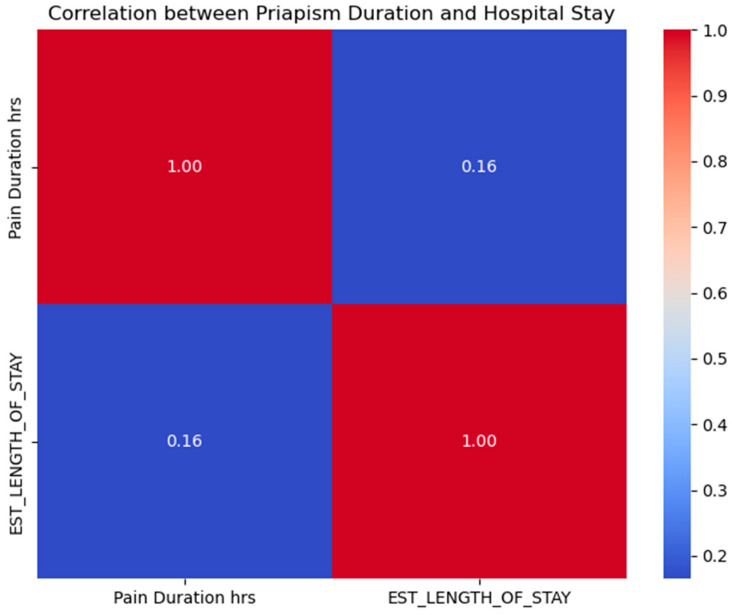
Correlation matrix between pain duration and the need for hospital admissions. Correlation matrix illustrating the weak positive relationship (r = 0.16) between priapism pain duration (hours) and the estimated length of hospital stay.

A significant association was found between the duration of priapism and hospital admission (*p* = 0.024), suggesting that longer episodes of priapism were more likely to result in hospital admission. The evaluation showed a slight positive correlation (rho = 0.165) between priapism episode duration (h) and hospital stay. This indicates that longer episodes of priapism may lead to a slightly prolonged duration of hospitalization, but other factors may contribute more. The length of hospital stay and treatment success (invasive or non-invasive) were also weakly positively correlated (rho = 0.068). This shows a weak inclination that a longer duration is linked with positive outcomes; however, the linkage is not firm as seen in Figure 6. Non-invasive treatments included penile aspiration and the intracavernous injection of phenylephrine. Invasive treatments involved shunt surgery for ischemic priapism and selective arterial embolization for non-ischemic cases.

Follow-up outcomes for sickle cell disease (SCD) patients with priapism were diverse, indicating a range of experiences and management strategies. 

Out of our total sample of 29 male patients, follow-up data were not available for 7 patients (24.14%), and 22 patients had follow-up data, and the mean follow-up duration was 18.5 months (range: 3–36 months). Patients who underwent bone marrow transplants had no further reported emergency room (ER) visits post-transplant.

## 4. Discussion

The present study was a retrospective analysis of the clinical features and treatment outcomes associated with priapism cases at the King Faisal Specialist Hospital & Research Centre in Riyadh, Saudi Arabia. The demographic patterns observed in most patients were from the central and southern regions, corresponding with the distribution of the population within Saudi Arabia. This observation is in line with the fact that sickle cell disease is more common in these regions. This implies a possible link between the regional load of sickle cell disease and the pattern of priapism cases, emphasizing the strong association between these two conditions [13].

### 4.1. Recurrent Priapism: A Call to Action on the Issue of Proactive Care

A high percentage of prior episodes of priapism (35.56%) highlight its recurrent nature and signify the necessity to ensure meticulous monitoring and active intervention strategies for such patients. This finding aligns with the global understanding of priapism as a potentially recurring condition [5,14]. Hence, further studies are needed to identify whether recurrent priapism is associated with delays in seeking treatment, unique intervention requirements, or worse outcomes.

On the contrary, prior priapism history and shunt surgery analysis revealed an unusual trend: 8.7% of the patients without previous priapism underwent emergency shunt surgery, but none of the patients with previous history did. This was contradictory to the findings discussed in the literature [7,9,12,15].

### 4.2. The Essential Role of Hematologic Collaboration

As predicted, our study revalidates the strong association between priapism and hematological disorders, especially sickle cell disease [10,11]. This emphasizes the critical requirement of teamwork between hematologists and urologists to provide the best care for this multifaceted patient population. The various long-term outcomes encountered among patients with sickle cell disease underline the need for personalized care based on the progress of the disease and the specific pathophysiology in each case [12,16].

### 4.3. Urological Conditions: A Possible Link

Since 15.56% of the patients had urological conditions (ED, BPH, and PD), the question arises whether these conditions might increase the susceptibility to priapism or share some common underlying risk factors. Exploring this possible interaction could lead to better preventive strategies and would greatly increase our understanding of the complex etiology of priapism [4].

### 4.4. Management of Priapism

Regarding the management of priapism, our study’s focus on non-surgical intervention in most cases is consistent with the current guidelines that advocate for conservative management initially [8]. The utilization of surgery in a limited number of cases in our study reflects the broader treatment landscape where surgical intervention is reserved for refractory cases or specific clinical scenarios [9].

### 4.5. Model Refinement and Classification Issues

The logistic regression model had the potential to predict ischemic priapism but had false positives at risk. Further refinement by considering other variables or alternative classification techniques could enhance its clinical usefulness.

The challenges in modeling stuttering priapism, likely due to sample size limitations, and the inability to predict non-ischemic priapism, highlight two key areas for future research.

### 4.6. Need for Larger Datasets

Modeling of less common priapism types and model performance improvement requires more extensive datasets.

### 4.7. Understanding Non-Ischemic Priapism

The unpredictable nature of non-ischemic priapism may indicate certain distinct subtypes, unaccounted factors (e.g., medications, rare conditions), or the need for other classification systems.

### 4.8. Individualized Care: The Road Ahead

Although our model has its own limitations, it implies that identifying factors predictive of ischemic priapism would allow for focused monitoring and prevention strategies. The diversity of stuttering and non-ischemic presentations requires personalized treatment approaches. Research oriented towards model refinement and the discovery of other specific risk factors will help customize care across the varied spectrum of priapism.

### 4.9. Comorbidities and Priapism Types

Our findings corroborate the strong link between sickle cell disease and ischemic priapism and underline the necessity for specific protocols and proactive follow up. The findings of associations between thalassemia, anemia, and priapism, particularly thalassemia and non-ischemic priapism, are observed; thus, further research is needed to understand the disease-specific mechanisms that could be related to priapism risk [17].

The broader trend of hypertension across the various types of priapism suggests that it could serve as a more generalized risk factor, perhaps due to its impact on vascular health. Additional studies on the interaction between the control of hypertension and prevention of priapism are imperative, particularly in the elderly populations. It is noteworthy that our study dealt with associations, so further research should examine the direct causality between comorbidities and specific priapism types.

### 4.10. Optimizing Triage, Treatment, and Pain Management

Our results question whether available triage protocols represent the severity and urgency of pain associated with priapism [18]. The major variations in pain duration between the types of priapism provide evidence of the subjectivity of pain, making it unreasonable to rely solely on the priapism type in determining optimal treatment. Doctors and patients in the ER show significant differences in pain intensity assessments, with doctors and nurses scoring lower than patients, possibly due to scaling discrepancies rather than actual pain perception differences [19]. Moreover, disparities in treatment success rates report a debate concerning the effect of triage designations on treatment outcomes, the role of patient factors in treatment response, and whether the order of interventions matters to patient outcomes [20].

Developing an extensive methodology for managing priapism is indispensable because it is essential to evaluate pain levels and classify priapism while assessing the severity of the condition. Future studies should explore the optimal timing and sequence of interventions based on the duration of pain to enhance patient outcomes and reduce the likelihood of long-term complications [21].

The complex nature of pain in priapism necessitates individualized treatment approaches. Pain experiences can vary significantly among patients, influenced by factors such as the duration of priapism, underlying causes, and individual characteristics. This variability underscores the need for comprehensive pain assessment in priapism management [19,20].

To address these diverse presentations and pain experiences, we propose a structured triage system for priapism management in emergency settings.

In the context of priapism management, we propose a three-level triage system: 2—Emergent, 3—Urgent, and 4—Less Urgent. Level 2—Emergent cases, seen within 10–15 min, include priapism lasting >4 h or with severe pain, requiring immediate intervention to prevent complications. Level 3—Urgent cases, seen within 30–60 min, involve priapism lasting 2–4 h or with moderate pain, needing prompt attention to prevent progression. Level 4—Less Urgent cases, seen within 1–2 h, encompass brief, recurring episodes or mild discomfort, requiring evaluation to prevent recurrence and address underlying causes. These triage levels should be applied in conjunction with clinical factors, priapism type, and individual patient considerations for optimal management in emergency settings.

This triage system, combined with a nuanced understanding of pain complexity, could enhance the management of priapism cases in Saudi Arabian emergency departments, potentially improving patient outcomes and resource allocation.

### 4.11. Penile Fibrosis as a Complication of Priapism

Penile fibrosis is a severe complication of prolonged ischemic priapism, resulting from smooth muscle necrosis and fibrosis of the corpora cavernosa [2,6,7]. Our study identified penile fibrosis as a dependent variable, underscoring its clinical significance. The incidence of penile fibrosis following priapism ranges from 25% to 80% in cases of prolonged ischemic priapism [6,7].

Broderick et al. elucidated the molecular mechanisms underlying penile fibrosis, demonstrating that prolonged hypoxia activates pro-fibrotic pathways, particularly TGF-β signaling [3]. This insight opens potential avenues for targeted therapeutic interventions. Prevention of penile fibrosis primarily relies on early intervention. Both the European Association of Urology (EAU) and the American Urological Association (AUA) guidelines emphasize the importance of prompt treatment within 24 h of onset to minimize the risk of permanent erectile dysfunction and fibrosis [6,7]. For patients who develop severe fibrosis, management options include timely penile prosthesis implantation, suggesting potential benefits for early implantation in selected cases [22].

### 4.12. Absence of Follow-Up Data

The absence of follow-up data for seven patients in our study sample underlines the necessity for strong follow-up protocols for monitoring outcomes and proactively addressing potential complications.

The findings of favorable outcomes in the recipients of bone marrow transplants indicate the important role of the procedure in reducing the likelihood of long-term priapism in appropriate sickle cell disease patients. These experiences emphasize the need for individual management focused on the unique trajectory of the disease, complications, and psychosocial support.

### 4.13. Limitations of the Study

The first and foremost limitation is the dataset size. The low number of just 26 priapism patients is due to a combination of multiple factors. The low overall incidence of priapism and the general difficulty of retrospective chart review, especially in a single center, certainly leads to a decreased sample size. Inconsistencies in formal documentation, absent data, and the lack of a standard follow-up term affected the completeness and presentation of the data. This limitation must be specifically tested, and future studies should investigate whether priapism cases were potentially managed in other departments and, hence, not documented in this study.

Moreover, working with several centers could overcome the limitations of the dataset size. These collaborative activities will enable access to a larger amount of data, and the outcomes will be more highly detailed in the description of priapism prevalence, its determinant factors, the course of the disease, and its consequences in populations of various types. Investigations that result in persistent and standard follow-up examinations are critical for appraising not only the direct impact of priapism on erectile function, penile change, and patient outcomes, but also the complete impact of the disorder.

## 5. Conclusions

This study provides valuable insights into the clinical presentation, characteristics, and management of priapism in a Saudi Arabian tertiary care setting. Our findings reveal a high recurrence rate of priapism, predominantly associated with hematological disorders, particularly sickle cell disease. This association highlights the chronic nature of priapism in patients with underlying blood disorders and aligns with the current literature reporting a strong link between sickle cell disease and recurrent priapism episodes.

The management of priapism in our study primarily involved non-surgical interventions, reflecting the current trend in priapism treatment. However, we observed a significant correlation between the duration of priapism episodes and the likelihood of hospital admissions. This finding suggests that prolonged episodes often require more intensive medical attention, potentially leading to increased healthcare utilization and costs.

Our results emphasize the need for tailored treatment approaches that consider underlying hematological disorders. For patients with sickle cell disease, this may necessitate a multidisciplinary approach combining urological interventions with hematological management strategies. Early identification and proactive management of at-risk patients could potentially reduce the frequency and severity of recurrent episodes.

The study also highlights the importance of institutional data in shaping clinical practice. In healthcare facilities where priapism is less frequently encountered, such as our tertiary care center in Saudi Arabia, these findings can guide the development of targeted screening programs and treatment protocols. This is particularly crucial given the potential for severe complications, including erectile dysfunction, if priapism is not managed promptly and effectively.

Future research directions should focus on the following:

1. Long-term follow-up studies to assess the efficacy of various treatment modalities in preventing recurrence.
2. Investigation of genetic factors that may predispose individuals with sickle cell disease to recurrent priapism.
3. Development and evaluation of preventive strategies for high-risk patients.
4. Assessment of quality-of-life impacts and psychosocial support needs for patients with recurrent priapism.

In conclusion, this study contributes to the understanding of priapism management in a Saudi Arabian tertiary care context, emphasizing the need for personalized care strategies, particularly for patients with underlying hematological disorders. By recognizing the chronic and recurrent nature of priapism in these patients, healthcare providers can work towards improving long-term outcomes and quality of life. These findings advocate for further research in healthcare settings where priapism is less prevalent, aiming to enhance the management and prevention of this challenging condition.

## Figures and Tables

**Table 1:** Age distribution analysis.

Age Group (Years)	Number of Patients	Percentage (95% CI)
0–20	2	6.9% (95% CI: 0.0–16.1%)
21–40	16	55.2% (95% CI: 37.1–73.3%)
41–60	9	31.0% (95% CI: 14.2–47.9%)
Over 60	2	6.9% (95% CI: 0.0–16.1%)

**Table 2:** Regional distribution of patients presented to KFSH.

Saudi Arabian Regions	Number of Patients	Percentage (95% CI)
Central	12	41.4% (95% CI: 23.5–59.3%)
Southern	8	27.6% (95% CI: 11.3–43.9%)
Northern	5	17.2% (95% CI: 3.5–31.0%)
Western	3	10.3% (95% CI: 0.0–21.4%)
Eastern	1	3.4% (95% CI: 0.0–10.1%)

**Table 3:** Concomitant pathologies and risk factors.

Category	Risk Factor	Number of Patients	Percentage (95% CI)
Hematological Disorders	Sickle cell disease	12	41.4% (95% CI: 23.5–59.3%)
	Thalassemia	2	6.9% (95% CI: 0.0–16.1%)
	Anemia	2	6.9% (95% CI: 0.0–16.1%)
Cardiovascular Risk Factors	Hypertension	4	13.8% (95% CI: 1.2–26.3%)
Cardiovascular Risk Factors	Diabetes	3	10.3% (95% CI: 0.0–21.4%)
Other	Peyronie’s disease	1	3.4% (95% CI: 0.0–10.1%)

**Table 4:** Number of patients and the amount of risk factors they had.

Risk Factor Count	Number of Patients	Percentage (95% CI)
Single risk factor	20	69.0% (95% CI: 52.1–85.8%)
Multiple risk factors	9	31.0% (95% CI: 14.2–47.9%)

**Table 5:** Multiple risk factors associated with our study sample.

Risk Factor Combination	Number of Patients	Percentage of Multiple Risk Factor Patients (95% CI)
Sickle cell disease + Hypertension	3	33.3% (95% CI: 2.5–64.1%)
Sickle cell disease + Diabetes	2	22.2% (95% CI: 0.0–49.4%)
Thalassemia + Anemia	2	22.2% (95% CI: 0.0–49.4%)
Hypertension + Diabetes	2	22.2% (95% CI: 0.0–49.4%)

**Table 6:** Types of priapism in our study population.

Priapism Type	Number of Patients	Percentage (95% CI)
Ischemic	20	69.0% (95% CI: 52.1–85.8%)
Non-ischemic	5	17.2% (95% CI: 3.5–31.0%)
Recurrent	4	13.8% (95% CI: 1.2–26.3%)

**Table 7:** Priapism management outcomes.

Category	Subcategory	Number of Patients	Percentage (95% CI)
Pain duration	<24 h	10	34.5% (17.2–51.8%)
	24–48 h	12	41.4% (23.5–59.3%)
	>48 h	7	24.1% (8.6–39.7%)
Management approach	Aspiration and intracorporeal injection	18	62.1% (44.4–79.7%)
	Shunt surgery	8	27.6% (11.3–43.9%)
	Penile prosthesis	1	3.4% (0.0–10.0%)
	Conservative management	2	6.9% (0.0–16.1%)
Outcome	Complete resolution	22	75.9% (60.3–91.4%)
	Partial resolution	5	17.2% (3.5–31.0%)
	No improvement	2	6.9% (0.0–16.1%)
Complication	Erectile dysfunction	6	20.7% (95% CI: 5.9–35.4%)
	Penile fibrosis	3	10.3% (95% CI: 0.0–21.4%)
	Urethral injury	1	3.4% (95% CI: 0.0–10.1%)

**Table 8:** Priapism types, mean symptoms duration, and treatment success.

Priapism Type	Patients (n)	Mean Symptom Duration (hours)	Treatment Success
Ischemic	22	39.49 ± 18.7	77.3%
Non-ischemic	3	28.33 ± 12.5	66.7%
Recurrent	4	72.17 ± 28.5	75.0%

**Table 9:** Logistic regression analysis results.

Predictor	B	SE	Wald	df	*p*	Odds Ratio
Pain Duration	0.05	0.02	6.25	1	0.012	1.05
Priapism Type (non-ischemic)	−1.79	0.98	3.33	1	0.068	0.17
Priapism Type (recurrent)	2.31	1.05	4.84	1	0.028	10.07
Constant	−3.42	1.01	11.47	1	0.001	0.03

Model Statistics: χ^2^(3) = 17.82, *p* < 0.001. Nagelkerke R^2^ = 0.521. Correct Classification = 79.3%.

**Table 10:** Treatment and outcomes of patients with sickle cell disease.

Treatment	Patients	Outcomes	Notes
Bone Marrow Transplantation	2	100% resolution of priapism episodes No further ER visits	Complete resolution in both patients.
Hydroxyurea Therapy	5	40% complete resolution 60% significant improvement Mean reduction in frequency: 68% (range: 50–85%) Mean time to reduction: 8.2 weeks (range: 6–12 weeks)	Dosage:15–20 mg/kg/day No major side effects reported.

## Data Availability

The raw data supporting the conclusions of this article will be made available by the authors on request.
